# Constructing Phylogenetic Networks Based on the Isomorphism of Datasets

**DOI:** 10.1155/2016/4236858

**Published:** 2016-07-28

**Authors:** Juan Wang, Zhibin Zhang, Yanjuan Li

**Affiliations:** ^1^School of Computer Science, Inner Mongolia University, Hohhot 010021, China; ^2^Department of Information and Computer Engineering, Northeast Forestry University, Harbin 150040, China

## Abstract

Constructing rooted phylogenetic networks from rooted phylogenetic trees has become an important problem in molecular evolution. So far, many methods have been presented in this area, in which most efficient methods are based on the incompatible graph, such as the CASS, the LNETWORK, and the BIMLR. This paper will research the commonness of the methods based on the incompatible graph, the relationship between incompatible graph and the phylogenetic network, and the topologies of incompatible graphs. We can find out all the simplest datasets for a topology *G* and construct a network for every dataset. For any one dataset *𝒞*, we can compute a network from the network representing the simplest dataset which is isomorphic to *𝒞*. This process will save more time for the algorithms when constructing networks.

## 1. Introduction

The evolutionary history of species is usually represented as a (rooted) phylogenetic tree, in which one species has only one parent. Actually, the evolution of species has caused reticulate events such as hybridizations, horizontal gene transfers, and recombinations [1–5], so species may have more than one parent. Then, the phylogenetic trees cannot describe well the evolutionary history of species. However, phylogenetic networks can represent the reticulate events, and they are a generalization of phylogenetic trees. Phylogenetic networks can also represent the conflicting evolution information that may be from different datasets or different trees [6–9].

Phylogenetic networks can be classified into unrooted [10–12] and rooted networks [4, 13–19]. An unrooted phylogenetic network is an unrooted graph whose leaves are bijectively labelled by the taxa. A rooted phylogenetic network is a rooted directed acyclic graph (DAG for short) whose leaves are bijectively labelled by taxa [20–22]. The rooted phylogenetic networks have been studied widely for representing the evolution of taxa, as evolution of species is inherently directed. The paper will study relevant properties of the rooted phylogenetic networks constructed from the rooted trees.

The algorithms constructing rooted phylogenetic networks from rooted phylogenetic trees are mainly classified into three types: the cluster network [17] based on the Hasse diagram; the galled network [16] based on the seed-growing algorithm; the Cass [23], the Lnetwork [24], and the BIMLR [25] based on the decomposition property of networks. In particular, the third type of methods (Cass, Lnetwork, and BIMLR) can construct more precise networks than the other methods. In the following, unless otherwise specified, we refer to rooted phylogenetic networks as networks.

**Figure 1:**
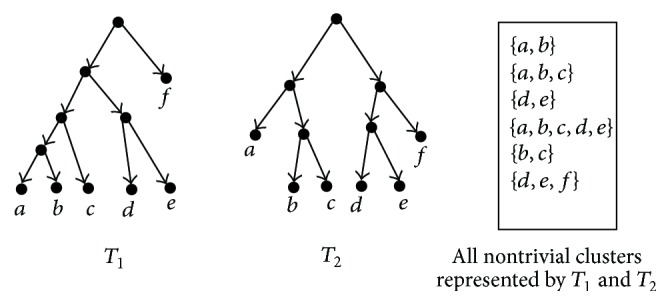
Two rooted phylogenetic trees *T*_1_ and *T*_2_.

Let *𝒳* be a set of taxa. A proper subset of *𝒳* (except for both *∅* and *𝒳*) is called a cluster. A cluster *C* is trivial if |*C*| = 1; otherwise, it is nontrivial. Let *T* be a rooted phylogenetic tree on *𝒳*; if there is an edge *e* = (*u*, *v*) in *T* such that the set of taxa which are descendants of *v* equals *C*, we say that *T* represents *C*.  Figure 1 shows two rooted phylogenetic trees *T*_1_ and *T*_2_ and all nontrivial clusters represented by *T*_1_ and *T*_2_. Here, all trivial clusters are not listed. Given a network *N* and a cluster *C*, when just connecting one incoming edge and disconnecting all other incoming edges for each reticulate node (i.e., its incoming edges >1), if there is a tree edge *e* = (*u*, *v*) (i.e., incoming edge of *v* ≤ 1) in *N* such that the set of taxa which are descendants of *v* equals *C*, we say that *N* represents *C* in the softwired sense. On the other hand, if there is a tree edge *e* = (*u*, *v*) in *N* such that the set of taxa which are descendants of *v* equals *C*, we say that *N* represents *C* in the hardwired sense.

The abovementioned three types of methods constructing networks are based on clusters; that is, they first compute all of the clusters represented by input trees and then construct a network representing all clusters in the softwired sense. In this process, the third type of methods (Cass, Lnetwork, and BIMLR) will recur to the incompatibility graph (will be discussed in the following). This paper will discuss the relationship between the incompatibility graphs and the constructed networks.

## 2. Preliminaries

A rooted phylogenetic network *N* = (*V*, *E*) on *𝒳* is a rooted DAG, and its leaves are bijectively labelled as *𝒳*. The indegree of a node *v* ∈ *V* is denoted by indeg(*v*). A node *v* with indeg(*v*) ≥ 2 is called a reticulate node, a node *v* with indeg(*v*) ≤ 1 is called a tree node, and, specially, the tree node with indegree 0 is the root node. The reticulation number in a network *N* = (*V*, *E*) is ∑_indeg(*v*)>0_(indeg(*v*) − 1) = |*E*| − |*V*| + 1.

Given a set of taxa *𝒳*, two clusters *C*_1_ and *C*_2_ on *𝒳* are called compatible, if they are disjoint or one contains the other; that is, *C*_1_∩*C*_2_ = *∅* or *C*_1_⊆*C*_2_ or *C*_2_⊆*C*_1_; otherwise, they are incompatible. Obviously, a trivial cluster and any one cluster are compatible. Given two incompatible clusters *C*_1_ and *C*_2_, *C*_1_∩*C*_2_ is called the incompatible taxa with respect to *C*_1_ and *C*_2_. A set of clusters *𝒞* on *𝒳* is called compatible, if *𝒞* is pairwise compatible; otherwise, it is incompatible. For a set of clusters *𝒞*, its incompatibility graph IG(*𝒞*) = (*V*, *E*) is an undirected graph with node set *V* = *C* and edge set *E*, where an edge connects two incompatible clusters.

Given a cluster set *𝒞* on *𝒳* and a subset *S* of *𝒳*, the result of removing all elements in *𝒳*∖*S* from each cluster in *𝒞* is called the restriction of *𝒞* to *S*, denoted by *𝒞*|_*S*_. If *S* (where |*S*| > 1) and any one cluster *C* ∈ *𝒞* are compatible and *𝒞*|_*S*_ is also compatible, then we say that *S* is an ST-set (Strict Tree Set) with respect to *𝒞*. If there are no other ST-sets containing *S* except itself, we say that *S* is maximal. For a maximal ST-set *S*, there is a subtree constructed by the set of clusters {*C*∣*C* ∈ *𝒞*, *C* ⊂ *S*} ∪ *S*.

For each maximal ST-set *S* with respect to *𝒞*, after collapsing it into a single taxon *S*, the result set is denoted as Collapse(*𝒞*). For example, *𝒞* = {{1,2}, {1,2, 3}, {3,4}}, {1,2} is the only maximal ST-set; then, Collapse(*𝒞*) = {{3,4}, {{1,2}, 3}}. Then, the taxa of Collapse(*𝒞*) are {{1,2}, 3,4}, denoted as *𝒳*(Collapse(*𝒞*)). A set of clusters *𝒞* is called the simplest if it has no maximal ST-set with respect to *𝒞*.

Let *𝒞* be a set of clusters on *𝒳* and let *N* be a network representing *𝒞*. Usually, a tree edge in *N* can represent more than one cluster in *𝒞* and a cluster in *𝒞* can be represented by more than one tree edge in *N*. A mapping *ϵ* is defined from *𝒞* to the set of tree edges of *N*, such that *ϵ*(*C*) is a tree edge of *N* that represents *C* for any one cluster *C* ∈ *𝒞*. A network *N* is decomposable with respect to *𝒞* if there exists a mapping *ϵ* : *𝒞* → *E*′ (*E*′ is the set of tree edges of *N*) such that

(i) for any two clusters *C*_1_, *C*_2_ ∈ *𝒞*, *C*_1_ and *C*_2_ lie in the same connected component of the incompatibility graph IG(*𝒞*) if and only if two tree edges *ϵ*(*C*_1_) and *ϵ*(*C*_2_) are contained in the same biconnected component of *N*.

Then, we also say that the network *N* has the decomposition property. The decomposition property makes the network constructed by an appropriate divide-and-conquer (DC for short) strategy; that is, it first constructs a subnetwork for each one connected component of the incompatibility graph and then merges all subnetworks into a whole network. Then, the constructed network is called DC network, and the algorithms are called DC algorithms. The paper [23] has proven the DC networks satisfying the decomposition property.

**Figure 2:**
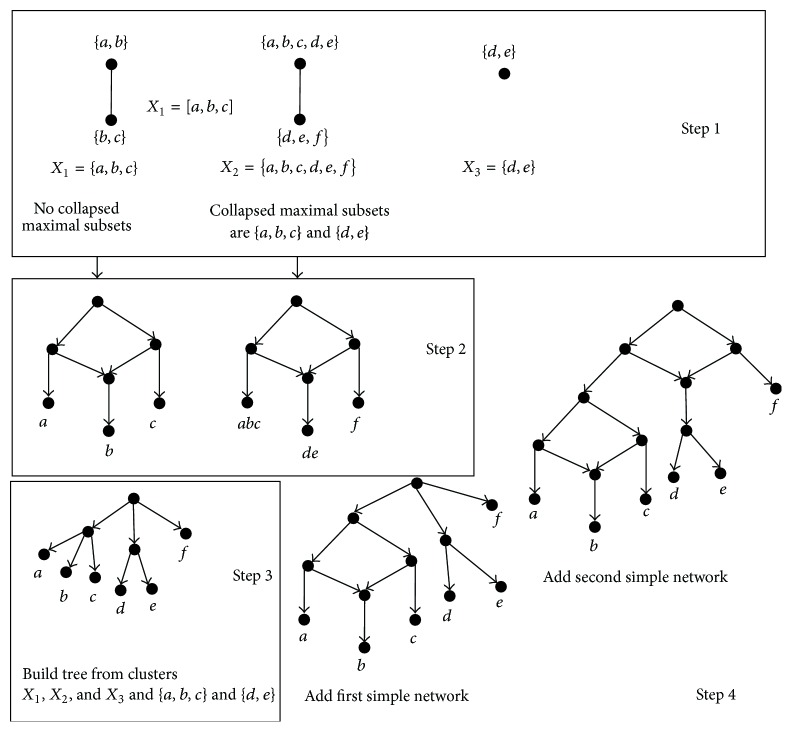
A network constructed by the DC algorithms for the set of clusters in Figure 1.

Given a set of clusters *𝒞*, the DC algorithms first compute the incompatibility graph IG(*𝒞*) and then compute the subnetwork for the result set after collapsing each one maximal ST-set into one taxon for each biconnected component of IG(*𝒞*); next, “decollapse,” that is, replace each leaf labelled by a maximal ST-set by a maximal subtree, and finally integrate those subnetworks into a final network. The paper [25] has proven that there exists a DC network *N* for any one set of clusters *𝒞*.  Figure 2 shows the construction process of the DC algorithms for the set of clusters in Figure 1, in which constructing subnetwork for each one connected component (i.e., Step 2) is crucial.

**Figure 3:**
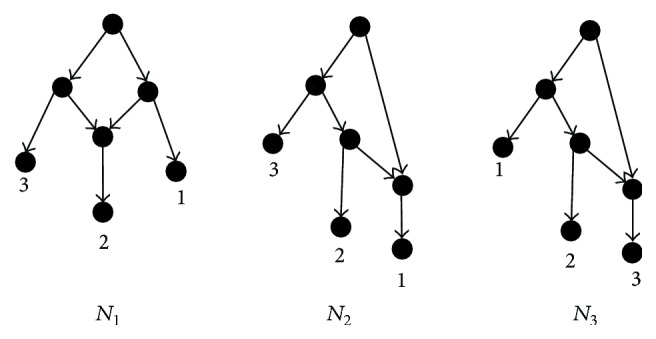
All networks constructed by the Cass for the set of clusters *𝒞* = {{1,2}, {2,3}}.

The Cass, the Lnetwork, and the BIMLR algorithms are the DC algorithms, which can construct the networks with fewer reticulations than other algorithms. The networks constructed by the BIMLR and the Lnetwork have fewer redundant clusters except for the input clusters than other available methods. When constructing phylogenetic networks, the BIMLR and the Lnetwork are faster than the Cass, and the constructed networks are more stable, that is, the difference between constructed networks for the same dataset when different input orders are used is smaller than the Cass. Figure 3 shows three networks constructed by the Cass for the same dataset with different input orders, while BIMLR and Lnetwork can construct only one network *N*_1_ for the dataset with different input orders [25].

## 3. Topologies of Incompatibility Graphs

**Definition 1 .**

Two networks *N*_1_ = (*V*_1_, *E*_1_) and *N*_2_ = (*V*_2_, *E*_2_) on *𝒳* are isomorphic if and only if there exists a bijection *H* from *V*_1_ to *V*_2_ such that

(i) (*u*, *v*) is an edge in *E*_1_ if and only if (*H*(*u*), *H*(*v*)) is an edge in *E*_2_;
(ii) the label of *w* is equal to the label of *H*(*w*) for any one leaf *w* ∈ *V*_1_.

Given two sets of clusters *𝒞*_1_ on *𝒳*_1_ and *𝒞*_2_ on *𝒳*_2_, let *𝒞*_1_′ and *𝒞*_2_′ be the results after collapsing all maximal ST-sets of *𝒞*_1_ and *𝒞*_2_, respectively, *𝒞*_1_′ on *𝒳*_1_′ and *𝒞*_2_′ on *𝒳*_2_′.

**Definition 2 .**

*𝒞*_1_ and *𝒞*_2_ are isomorphic, if and only if there is a bijection *G* from *𝒳*_1_′ to *𝒳*_2_′ such that

(i) *a* and *b* are in the same cluster *C*_1_ ∈ *𝒞*_1_′ if and only if *G*(*a*) and *G*(*b*) are in the same cluster *C*_2_ ∈ *𝒞*_2_′.

By Definition 2, we have that the isomorphism of the cluster sets is an equivalence relation; that is, it is reflexive, symmetric, and transitive.

**Lemma 3 .**

Given a DC network *N* representing the set of clusters *𝒞*, then any one maximal ST-set with respect to *𝒞* is a maximal subtree in *N*.

**Proof**

From the constructing process of DC networks, this conclusion is obvious.

**Lemma 4 .**

Let *𝒞*_1_ and *𝒞*_2_ be two sets of clusters on *𝒳*_1_ and *𝒳*_2_, respectively. *𝒞*_1_ and *𝒞*_2_ are isomorphic. There exists a DC network *N*_1_ representing *𝒞*_1_ if and only if there exists a DC network *N*_2_ representing *𝒞*_2_.

**Proof**

There must exist a DC network *N*_1_ for *𝒞*_1_. Given a tree edge *e* = (*u*, *v*), the subtree of the root *v* in *N*_1_ is a maximal subtree if and only if the set of taxa *S* is a maximal ST-set with respect to *𝒞*_1_, where the taxa in *S* are labels of leaves which are descendants of *v*. Replace each maximal subtree of *N*_1_ by a node, and then denote the result network as *N*_1_′. Obviously, *N*_1_′ represents the set of clusters *𝒞*_1_′. From Definition 2, there exists a bijection *G* from *𝒳*_1_′ to *𝒳*_2_′ such that *a* and *b* are in the same cluster *C*_1_ ∈ *𝒞*_1_′ if and only if *G*(*a*) and *G*(*b*) are in the same cluster *C*_2_ ∈ *𝒞*_2_′.

Then, we can obtain a network *N*_2_′ from *N*_1_′ by replacing each one taxon *a* in *𝒳*_1_′ by *G*(*a*) in *𝒳*_2_′. Obviously, *N*_2_′ represents *𝒞*_2_′. Finally, we replace each leaf labelled by a maximal ST-set with respect to *𝒞*_2_ in *N*_2_′ by a maximal subtree, and the result network is denoted as *N*_2_ which represents *𝒞*_2_.

For two isomorphic sets of clusters *𝒞*_1_ and *𝒞*_2_, let *N*_1_ be a DC network representing *𝒞*_1_.  Lemma 4 tells us that there is a DC network *N*_2_ representing *𝒞*_2_, which can be obtained from *N*_1_.

**Lemma 5 .**

Let *ℭ* = {*𝒞*∣*𝒞* *is* *a* *set* *of* *clusters*}, where *IG*(*𝒞*) is a biconnected component with two nodes. Then, any one element *𝒞* in *ℭ* is isomorphic to *𝒞*_0_ = {{1,2}, {2,3}}.

**Proof**

Any one element *𝒞* ∈ *ℭ* has two incompatible clusters. Let *𝒞*_1_ = {*C*_11_, *C*_12_} and *𝒞*_2_ = {*C*_21_, *C*_22_} be two sets of clusters in *ℭ*, where *C*_11_ and *C*_12_ are incompatible and *C*_21_ and *C*_22_ are incompatible. Let *A*_1_ = *C*_11_∩*C*_12_ be the incompatible taxa with respect to *C*_11_ and *C*_12_, and let *A*_2_ = *C*_21_∩*C*_22_ be the incompatible taxa with respect to *C*_21_ and *C*_22_. Let *B*_11_ = *C*_11_∖*A*_1_, *B*_12_ = *C*_12_∖*A*_1_, *B*_21_ = *C*_21_∖*A*_2_, and *B*_22_ = *C*_22_∖*A*_2_; then, *𝒞*_1_ = {{*B*_11_, *A*_1_}, {*B*_12_, *A*_1_}} and *𝒞*_2_ = {{*B*_21_, *A*_2_}, {*B*_22_, *A*_2_}}.

Each one of *B*_11_, *A*_1_, *B*_12_, *B*_21_, *A*_2_, and *B*_22_ is a maximal ST-set if it contains more than one taxon; then, we can collapse it into one taxon which is also denoted by itself. Denote the set of clusters after collapsing all maximal ST-sets as *𝒞*_1_′ and *𝒞*_2_′. Obviously, there is a bijection *G* from *𝒳*_1_′ = {*B*_11_, *A*_1_, *B*_12_} to *𝒳*_2_′ = {*B*_21_, *A*_2_, *B*_22_}, and any two taxa *a*, *b* ∈ *𝒳*_1_′ are in the same cluster in *𝒞*_1_′ if and only if *G*(*a*) and *G*(*b*) are in the same cluster in *𝒞*_2_′. Hence, *𝒞*_1_ and *𝒞*_2_ are isomorphic. Accordingly, any one set of clusters *𝒞* ∈ *ℭ* is isomorphic to *𝒞*_0_ = {{1,2}, {2,3}} because *𝒞*_0_ ∈ *ℭ*.

For a cluster set *𝒞*, there may be several cluster sets isomorphic to *𝒞*, but the simplest set of clusters isomorphic to *𝒞* is only one, denoted as *𝒞*_0_. Let *N*_0_ be the DC network representing *𝒞*_0_. Then, we can obtain a DC network representing *𝒞* from *N*_0_. Lemmas 4 and 5 show there is a DC network for any one set of clusters whose incompatible graph is a biconnected component with two nodes, and it is obtained from the network *N*_0_ (see Figure 3) representing *𝒞*_0_.

**Lemma 6 .**

**Figure 4:**
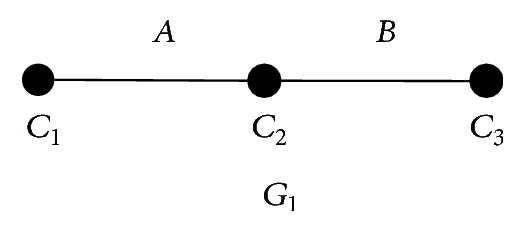
The topology of the linear biconnected component with three nodes.

Let *ℭ* = {*𝒞*∣*𝒞* *is* *a* *set* *of* *clusters*}, where *IG*(*𝒞*) is a linear biconnected component with three nodes (see Figure 4). Let *𝒞*_1_ = {{1,3}, {1,2}, {1,3, 4}}, *𝒞*_2_ = {{1,3}, {1,2, 4}, {1,2, 3}}, *𝒞*_3_ = {{1,2}, {2,3}, {3,4}}, and *𝒞*_4_ = {{1,2}, {2,3, 5}, {3,4}}. Then, any one set of clusters *𝒞*  (*𝒞* ∈ *ℭ*) is isomorphic to one of *𝒞*_1_, *𝒞*_2_, *𝒞*_3_, and *𝒞*_4_.

**Proof**

Figure 4 shows the topology of the linear biconnected component with three nodes. *𝒞*_*i*_ is the simplest set of clusters, and its incompatible graph is the topology in Figure 4. Next, we will prove that *𝒞*_*i*_  (1 ≤ *i* ≤ 4) are all simplest sets of clusters for the topology in Figure 4.

Any one set of clusters in *ℭ* has three clusters denoted as *C*_1_, *C*_2_, and *C*_3_. Let *A* be the incompatible taxa with respect to *C*_1_ and *C*_2_, and let *B* be the incompatible taxa with respect to *C*_2_ and *C*_3_; then *A* and *B* have the following cases: (i) *A* = *B*; (ii) *A* ⊂ *B*; (iii) *B* ⊂ *A*; (iv) *A*∩*B* = *∅*; (v) *A*∩*B* ≠ *∅*, *A*⊈*B* and *B*⊈*A*.

*(i) A* = *B*. Since there is no edge between *C*_1_ and *C*_3_, *C*_1_ and *C*_3_ are compatible; that is, *C*_1_∩*C*_3_ = *∅*, or *C*_1_⊆*C*_3_, or *C*_3_⊆*C*_1_. Because *A*⊆*C*_1_ and *A*⊆*C*_3_, we have that *C*_1_∩*C*_3_ ≠ *∅*. Therefore, *C*_1_⊆*C*_3_ or *C*_3_⊆*C*_1_. Then, we have the simplest set of clusters *𝒞*_1_ = {{1,3}, {1,2}, {1,3, 4}}, and any one set of clusters in this case is isomorphic to *𝒞*_1_.

*(ii) A* ⊂ *B*. Assume that *B* = {*A*, *B*_0_}. It is similar to the case (i), and we have that *C*_1_⊆*C*_3_. Then, the simplest set of clusters is *𝒞*_2_ = {{1,3}, {1,2, 4}, {1,2, 3}}, and any one set of clusters in this case is isomorphic to *𝒞*_1_.

*(iii) B* ⊂ *A*. This case is similar to case (ii). The sets of clusters are in case (ii) if and only if they are in case (iii). Hence, any one set of clusters in case (iii) and *𝒞*_2_ are isomorphic.

*(iv) A*∩*B* = *∅*. Then, *C*_1_∩*C*_3_ = *∅*. We have that |*A*| = 1 and |*B*| = 1 in the simplest set of clusters, since they can be collapsed if |*A*| ≥ 2 or |*B*| ≥ 2. Assume that *C*_1_ = {*A*, *B*_1_} and *C*_3_ = {*B*, *B*_2_}. We have that |*B*_1_| = 1 and |*B*_2_| = 1 in the simplest set of clusters, since they can be collapsed if |*B*_1_| ≥ 2 or |*B*_2_| ≥ 2. Then, |*C*_1_| = 2 and |*C*_3_| = 2 in the simplest set of clusters. *𝒞*_3_ = {{1,2}, {2,3}, {3,4}} and *𝒞*_4_ = {{1,2}, {2,3, 5}, {3,4}} are the simplest sets of clusters in this case. Therefore, any one set of clusters in this case is isomorphic to *𝒞*_3_ or *𝒞*_4_.

*(v) A*∩*B* ≠ *∅*, *A*⊈*B and B*⊈*A*. Let *A* = {*A*_0_, *A*_1_} and *B* = {*A*_1_, *B*_0_}, where *A*_0_, *A*_1_, and *B*_0_ are not empty. We have {*A*_0_, *A*_1_, *B*_0_}⊆*C*_2_, and *C*_1_⊆*C*_3_ or *C*_3_⊆*C*_1_. If *C*_1_⊆*C*_3_, then *A*_1_⊆*C*_3_. So *A*_1_⊆*B*, which contradicts the case that *A*⊈*B*. Similarly, we can get the contradiction when *C*_3_⊆*C*_1_. Thus, there exists no set of clusters in this case.

**Figure 5:**
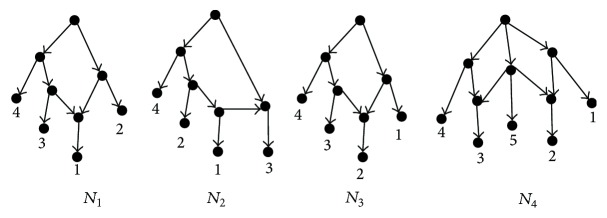
The DC networks for all simplest cluster sets whose incompatible graphs are topologies in Figure 4.

Figure 5 shows the DC networks for the simplest sets of clusters *𝒞*_1_, *𝒞*_2_, *𝒞*_3_, and *𝒞*_4_, respectively.

**Lemma 7 .**

**Figure 6:**
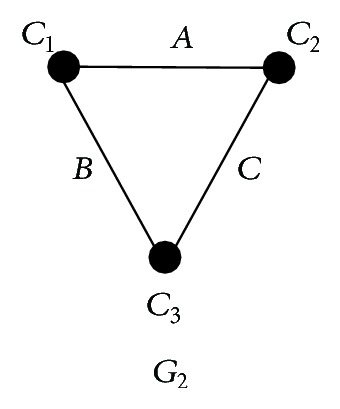
The topology of the nonlinear biconnected component with three nodes.

Let *ℭ* = {*𝒞*∣*𝒞* *is* *a* *set* *of* *clusters*}, where *IG*(*𝒞*) is a nonlinear biconnected component with three nodes (see Figure 6). Let *𝒞*_1_ = {{1,3}, {1,2, 4}, {1,2, 5}}, *𝒞*_2_ = {{1,2}, {1,3}, {1,4}}, *𝒞*_3_ = {{1,2, 4}, {1,3, 5}, {1,2, 3}}, *𝒞*_4_ = {{1,2, 4}, {1,3, 5}, {1,2, 3,6}}, *𝒞*_5_ = {{1,2}, {2,3}, {1,3}}, *𝒞*_6_ = {{1,2, 4}, {1,3}, {2,3}}, *𝒞*_7_ = {{1,2, 4}, {1,3, 5}, {2,3}}, *𝒞*_8_ = {{1,2, 4}, {1,3, 5}, {2,3, 6}}, *𝒞*_9_ = {{1,2, 3}, {1,2, 4}, {1,3, 4}}, *𝒞*_10_ = {{1,2, 3,5}, {1,2, 4}, {1,3, 4}}, *𝒞*_11_ = {{1,2, 3,5}, {1,2, 4,6}, {1,3, 4}}, and *𝒞*_12_ = {{1,2, 3,5}, {1,2, 4,6}, {1,3, 4,7}}. Then, any one set of clusters in *ℭ* is isomorphic to one of *𝒞*_*i*_  (1 ≤ *i* ≤ 12).

**Proof**

Figure 6 shows the topology of the nonlinear biconnected component with three nodes. Here, *C*_1_, *C*_2_, and *C*_3_ are the clusters, and *A*, *B*, and *C* are the incompatible taxa corresponding to them. All cases are as follows: (i) *A* = *B*; then, *A*⊆*C* or *A* = *C*; (ii) *A* ⊂ *B*; then, *A* ⊂ *C*, and *C*∩*B* = *A*; (iii) *A*∩*B* = *∅*; then, *A*∩*C* = *∅* and *B*∩*C* = *∅*; (iv) *A*∩*B* ≠ *∅*, *A*⊈*B*, *B*⊈*A*; then, *A*∩*C* ≠ *∅* and *B*∩*C* ≠ *∅*.

*(i) A* = *B*. If *A*⊆*C*, then *A*⊆*C*_1_, *C*⊆*C*_2_, and *C*⊆*C*_3_. We have |*A* | = 1 in the simplest set of clusters; otherwise, *A* can be collapsed into one taxon. Similarly, we have |*C* | = 2 in the simplest set of clusters. Let *A* = {1} and *C* = {1,2}; then, we can obtain the only simplest set of clusters *𝒞*_1_ = {{1,3}, {1,2, 4}, {1,2, 5}}. Any one set of clusters meeting this case will be isomorphic to *𝒞*_1_.

If *A* = *C*, then *A* = *B* = *C*. There is |*A* | = 1 in the simplest set of clusters; otherwise, *A* can be collapsed into one taxon. Let *A* = *B* = *C* = {1}; then, we can obtain the only simplest set of clusters *𝒞*_2_ = {{1,2}, {1,3}, {1,4}}. Any one set of clusters in this case will be isomorphic to *𝒞*_2_.

*(ii) A* ⊂ *B*, *A* ⊂ *C, and C*∩*B* = *A*. Then, we can obtain the simplest sets of clusters *𝒞*_3_ = {{1,2, 4}, {1,3, 5}, {1,2, 3}} and *𝒞*_4_ = {{1,2, 4}, {1,3, 5}, {1,2, 3,6}}. Any one set of clusters in this case will be isomorphic to *𝒞*_3_ or *𝒞*_4_.

*(iii) A*∩*B* = *∅; then, A*∩*C* = *∅ and B*∩*C* = *∅*. Then, we can obtain the simplest sets of clusters *𝒞*_5_ = {{1,2}, {2,3}, {1,3}} and *𝒞*_6_ = {{1,2, 4}, {1,3}, {2,3}} and *𝒞*_7_ = {{1,2, 4}, {1,3, 5}, {2,3}} and *𝒞*_8_ = {{1,2, 4}, {1,3, 5}, {2,3, 6}}. Any one set of clusters in this case will be isomorphic to one of *𝒞*_5_, *𝒞*_6_, *𝒞*_7_, and *𝒞*_8_.

*(iv) A*∩*B* ≠ *∅*, *A*⊈*B*, *B*⊈*A; then, A*∩*C* ≠ *∅ and B*∩*C* ≠ *∅*. Let *A*∩*B* = *A*_0_; then, *A*∩*C* = *A*_0_ and *B*∩*C* = *A*_0_. We have |*A*_0_| = 1 in the simplest set of clusters; otherwise, *A*_0_ can be collapsed into one taxon. Let *A*_0_ = {1}. Then, *A* = {1,2}, *B* = {1,3}, and *C* = {1,4}. For the first case, we can obtain the simplest sets of clusters *𝒞*_9_ = {{1,2, 3}, {1,2, 4}, {1,3, 4}} and *𝒞*_10_ = {{1,2, 3,5}, {1,2, 4}, {1,3, 4}} and *𝒞*_11_ = {{1,2, 3,5}, {1,2, 4,6}, {1,3, 4}} and *𝒞*_12_ = {{1,2, 3,5}, {1,2, 4,6}, {1,3, 4,7}}. Any one set of clusters in this case will be isomorphic to one of them.

**Figure 7:**
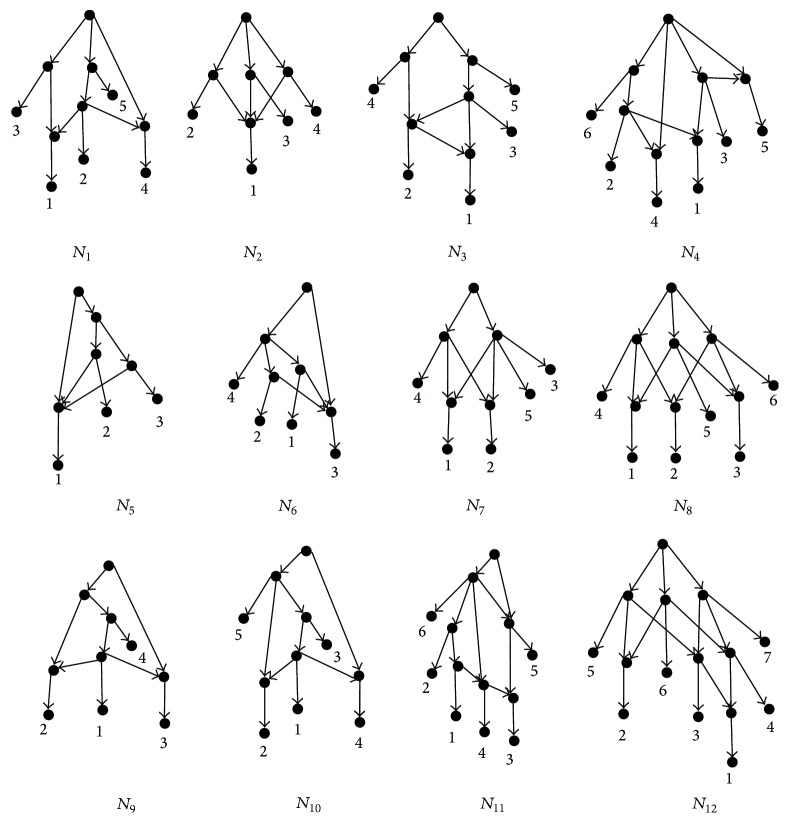
The DC networks for all simplest cluster sets whose incompatible graphs are topologies in Figure 6.

Figure 7 shows the DC networks for the simplest sets of clusters *𝒞*_*i*_  (1 ≤ *i* ≤ 12), respectively. Lemmas 5, 6, and 7 compute all simplest sets of clusters, whose incompatible graphs are the biconnected components with two nodes or three nodes. Figures 6 and 7 show the DC networks constructed by the BIMLR algorithm for all simplest sets of clusters; then, the DC network for a set of clusters *𝒞* can be obtained from the DC network representing the simplest set of clusters which is isomorphic to *𝒞*; that is, it does not need to be constructed once again. This conclusion is very important to the construction of networks.

## 4. Conclusion

This paper computes all simplest sets of clusters for the topologies of incompatible graph with two nodes and three nodes. We can construct the DC networks for those simplest sets of clusters and save them. When constructing DC networks for any one set of clusters *𝒞*, algorithms only need to read the DC network *N*_0_ of the simplest set of clusters isomorphic to *𝒞* and then compute the DC network for *𝒞* from *N*_0_ by replacing labels of leaves in *N*_0_ by the taxa in *𝒞*, which will save more time for the algorithms.

We will compute the simplest sets of clusters for more topologies of incompatible graph in the future.
